# Vincristine induced neurotoxicity in children who underwent chemotherapy for acute lymphoblastic leukemia and Wilms tumor

**DOI:** 10.12669/pjms.37.5.4169

**Published:** 2021

**Authors:** Muhammad Kamran Adil, Zulfiqar Ali, Uzma Arshad, Usman Fawad

**Affiliations:** 1 Dr. Muhammad Kamran Adil Resident Paeds Hematology, Oncology, Children Hospital & the Institute of Child Health, Multan, Pakistan; 2 Dr. Zulfiqar Ali Assistant Professor, Paeds Hematology, Oncology, Children Hospital & the Institute of Child Health, Multan, Pakistan; 3 Dr. Uzma Arshad Assistant Professor, Community Medicine, Multan Medical & Dental College, Multan, Pakistan; 4 Dr. Usman Fawad Resident Paeds Hematology, Oncology, Children Hospital & the Institute of Child Health, Multan, Pakistan

**Keywords:** Vincristine, Neurotoxicity, Children, Chemotherapy, Acute Lymphoblastic Leukemia, Wilms tumor

## Abstract

**Background & Objectives:**

Vincristine has been used as chemotherapeutic agent for many decades. It implements its function by inhibiting the duplication of tumor cells by destroying the DNA. However, like all other drugs, its administration is not without any side effects. The most important of these are being the neurotoxic side effects. This study evaluated the degree of neurotoxicity induced by vincristine in children who underwent chemotherapy for acute lymphoblastic leukemia and Wilms tumor.

**Methods:**

A quasi experimental study was conducted at Children Hospital & the Institute of Child Health, Multan from January 2020 to October 2020 after taking informed written consent. In this study, 150 children of age group 1 – 12 years with pathological confirmation of acute lymphoblastic leukemia and Wilms tumor who had undergone a chemotherapy protocol including at least four consecutive weekly Vincristine injections were included, using probability consecutive sampling technique. Neurological examination was conducted on them on weekly basis.

**Results:**

There were 150 patients,90(60%) males and 60(40%) females with mean age of (5.5±2.2). Diminished patellar and Achilles tendon reflexes were seen in 48% and 52% of patients. Muscular weakness was seen in 60% of patients. Other side effects like hoarseness, jaw pain, constipation and petosis were observed in 10%, 8%,40% and 10% of patients respectively. Frequency of side effects was equally observed in both sexes and it was more among age group older than five years (p= 0.01).

**Conclusion:**

Vincristine regimen produces some neurotoxic side effects in children but nearly all of these are of mild to moderate in nature.

## INTRODUCTION

Acute lymphoblastic leukemia is a type of cancer characterized by clonal expansion and arrest at a particular stage of usual lymphoid hematopoiesis and it mainly affects the children. Most of the cases are seen in age group 2.5-5 years.^1^ Vincristine also known as leurocristine, administered intravenously is the drug of choice^2^ in the treatment of acute lymphoblastic leukemia, lymphoid blast crises of chronic myeloid leukemia, Hodgkin and non-Hodgkin lymphoma as confirmed by the United States Food and Drug Administration. Vincristine can also be used for several other CNS tumors.^3^ This drug was recognized for the first time in 1961, a vinca alkaloid which is generated from the Madagascar periwinkle Catharanthusroseus. There are various derivatives of Catharanthus like Vinblastine, Vincristine, Vindesine and a new derivative of Vincristine, “Vinepidine”. Vincristine is also included in List of Essential medicines by WHO.^4^ Vincristine lead to the formation of microtubules as well as blocks the glutamic acid utilization.^5^ Vincristine effects on multiple systems of body as a part of its therapeutic side effects,^6^ and among them neurotoxicity is the remarkable side effect and this effect is mainly dose dependand.^7^

The symptoms of this are quite progressive in nature that starts in hands and feet and afterwards progressing to arms and legs. Foot drop is considered as first symptom of peripheral neuropathy. Persons having family history regarding foot drop or Charcot Marie – Tooth disease should be discouraged to take vincristine as it leads to aggravation to previously occurring neurological diseases. Its administration also requires great care as unintentional administration into the spinal canal is extremely fatal with mortality rate of up to 100%. Some patients may develop encephalopathy with ascending paralysis and after timely management only few patients can survive. Similarly, excessive use of vincristine may results in drug resistance.^8^ Vincristine induced peripheral neuropathy pose a challenge to pediatric oncologists in terms of its detection and monitoring in pediatric patients. In these patients it can badly effects the quality of life.^8^,^9^ Depending on degree of side effects, the drug administration can be interfered with or may cause its discontinuation that may effect the treatment of malignancy. So in patients on vincristine multiple aspects should be considered and among them patient’s neurological function have major importance. The current study was planned to determine the extent of neurotoxicity induced by administration of four weekly injection of VCR in children undergoing chemotherapy for ALL and Wilms tumor.

## METHODS

A quasi experimental study was carried out at hematology oncology ward of Children Hospital & Institute of Child Health, Multan for duration of ten months from January to October 2020 after obtaining the Institutional and Ethical Approval (IRB-2019, No-436). One hundred and fifty children of age group between one to 12 years of age with pathological confirmation of ALL and Wilms tumor were included in the study using probability consecutive sampling technique. Of these 140 children had ALL and 10 patients were of Wilms tumor. children suffering from any other neurological disorder, CNS leukemia, previous history of chemotherapy or relapsed disease were excluded from the study. Patients with Acute lymphoblastic leukemia were divided into two groups i.e. Standard and High risk group. Standard group includes patients having age>1 year and <10 year, WBC <50×10 9 /L, no testicular disease, B cell phenotypes and no high risk cytogenetics. High risk group have one of the following features like age ≥ 10 years, WBC ≥ 50 × 10^9^, CNS disease, T cell phenotype, testicular involvement with leukemia and high risk cytogenetics. The chemotherapeutic regime included, Dexamethasone (6mg /m^2^/day) for three weeks. Four weekly injections of VCR (1.5mg/m^2^/max-2mg).9 injections of *E.coli* Asparaginase 6000 units/m^2^/dose. Three intrathecal injections of Methotrexate. Four additional injections of Daunorubicin 25mg/m^2^/dose for high risk patients.

Patients with Wilms tumor having localized disease were treated according to SIOP 2001 protocol i.e. Four weekly injections of vincristine (1.5 mg/m^2^/max-2mg) and Actinomycin (45µg/kg/dose). All patients were examined on days 0, 14, 28, 42, 56, and 70 for the onset of side effects. Patellar and Achilles tendon reflexes along with weakness of lower extremity muscles were checked. Other complaints like jaw pain, abdominal pain, constipation and urinary retention were also considered. Data was then analyzed using SPSS Version 20. Stratification with respect to age was done. Post stratification chi square test was applied by taking p ≤ 0.05 as significant.

## RESULTS

Total study population was 150 with mean age 5.5 and SD ±2.2. Out of these 90(60%) were males and 60(40%) were females. Patellar and Achilles reflexes were diminished in 72(48%) and 78(52%) of patients respectively. Muscular weakness in lower extremities was seen in 90(60%) of patients. Ptosis was observed in 15(10%) of patients. Other neurological side effects documented as, jaw pain in 12(8%), hoarseness in 15(10%), and constipation in 60(40%) of patients. No patient showed foot drop, urinary retention and facial nerve palsy. Both sexes have same rate of neurotoxic side effects of vincristine. Side effects were mostly seen in children older than five years (p = 0.01)

**Table-I:** Demographic profile of participants.

Sex	Male	90 (60%)
	Female	60(40%)
Age Year	1-3	13 (8.67%)
	4-6	30 (20%)
	7-9	62 (41.33%)
	10-12	45 (30%)
Diagnostic	ALL	140
	Wilms tumor	10

**Table-II:** Appearance of sign & symptoms of Neurotoxity.

Symptom	Time of appearance in days					

	14	28	42	56	70	Total
Decreased Achilles reflex	4 (5.13%)	23 (29.47%)	31(39.75%)	8 (10.26%)	12 (15.39%)	78 (52%)
Decreased patellar reflex	..2(2.78%)	16 (22.22%)	23 (31.94%)	20(27.78%)	11 (15.28%)	72 (48%)
Muscles weakness	-	30 (33.33%)	15 (16.67%)	45 (50%)	-	90 (60%)
Ptosis	3 (20%)	2 (13.33%)	6 (40%)	4 (26.67%)	-	15 (10%)
Hoarseness	2(13.33%)	4 (26.67%)	-	8 (53.33%)	1 (6.67%)	15 (10%)
Foot drop	-	-	-	-	-	-
Urinary retention	-	-	-	-	-	-
Facial nerve palsy	-	-	-	-	-	-
Constipation	20(33.33%)	10 (16.67%)	7 (11.67%)	23(38.33%)	-	60 (40%)
Jaw pain	10(83.33%)	2 (16.67%)	-	-	-	12 (8%)

## DISCUSSION

Vincristine also termed as leucocristine, a chemotherapeutic medicine known to treat a wide range of cancers, the notable being the acute lymphoblastic leukemia, acute myeloid leukemia, Hodgkin’s disease and neuroblastoma. Vincristine regime when used for chemotherapeutic purpose in patients is responsible for multiple side effects.

In our study, all the cases were followed for whole length of treatment. But there is a study in which 15 patients were lost to follow up, may be due to poverty, and lack of awareness.^10^ In our study, Vincristine showed different side effects in patients. Most remarkable side effects were being the diminished tendon reflexes and muscular weakness. Similar findings are also seen in other studies on Vincristine.^11^ No patient showed signs of foot drop, urinary retention and facial nerve palsy in this study. At the start of our study, neurological examination of all the patients was done in order to exclude any bias and it was normal. This shows that the neurological signs seen in patients are because of drug and not because of disease.

The neurological signs and symptoms seen in patients were of mild to moderate in nature in our study. In contrast to this, there is a case study in Ocotal that showed severe type of lower limb motor axonal polyneuropathy effecting the lower limbs in a seven year old male patient with vincristine that occurred about four weeks after starting the drug.^12^ The child also had history of joint pain from previous two years.

In our study neuropathic side effects were seen mostly in children more than five years of age showing younger patients are less susceptible to vincristine side effects. Other studies also showed association of age with side effects.^13^ Gastrointestinal side effects were also seen in some of our patients, but luckily all of these are of minor nature but there are studies in which gastrointestinal related side effects may hamper the cancer management. A study conducted in Australia showed 80% of patients having diarrhea after administration of chemotherapy.^14^ Similarly, there is another case report in which patient developed severe constipation after administration of vincristine regime that could not be relieved by multiple laxative drugs for constipation.^15^

The fact about the onset of vincristine induced side effects is quite unclear. Some studies demonstrate it to be genetically linked.^16^ A meta analysis on cohorts showed two single nucleotide polymorphisms rs1045644 and rs7963521 were mainly responsible for neuropathy.^17^ Some demonstrate it to be dose linked.^12^ Some argued that neuropathy may be due to cumulative toxicity resulting from administration of vincristine for a prolonged period of time and by decreasing in drug dosage, these adverse effects may diminished or totally vanished. But whatever the cause of its side effects, vincristine has saved hundreds and thousands of lives.

Our study can be utilized to provide knowledge to the patients and their attendants regarding commonly occurring vincristine induced neurotoxic side effects. Patients and their attendants should have knowledge about these side effect. As acute lymphoblastic leukemia is mostly encountered in kids of 2-3 years of age that are unable to describe these symptoms, in this scenario doctors and patient’s attendants should play an active role in reporting the side effects as soon as possible.

## CONCLUSION

Vincristine is widely used as chemotherapeutic agent in cancer patients. It produces side effects which are well tolerable by the patients. Vincristine is notorious for its peripheral neurotoxic side effects which are of mild to moderate in nature.

### Author’s Contribution:

**MKA:** Conceived the study, data collection and analysis, interpretation, supervision and responsible for integrity of study.

**ZA:** Supervision of the project.

**UA:** Data analysis, interpretation, literature search and drafting.

**UF:** Data collection, Literature search and analysis.
